# Structural, Electronic and Vibrational Properties of B_24_N_24_ Nanocapsules: Novel Anodes for Magnesium Batteries

**DOI:** 10.3390/nano14030271

**Published:** 2024-01-26

**Authors:** Domenico Corona, Francesco Buonocore, Friedhelm Bechstedt, Massimo Celino, Olivia Pulci

**Affiliations:** 1 Department of Physics, University of Rome Tor Vergata and INFN, Via della Ricerca Scientifica 1, 00133 Rome, Italy; 2 Energy Technologies and Renewable Sources (TERIN) Department, Italian National Agency for New Technologies, Energy and Sustainable Economic Development (ENEA), Casaccia Research Centre, 00123 Rome, Italy; francesco.buonocore@enea.it (F.B.); massimo.celino@enea.it (M.C.); 3 Institut für Festkörpertheorie und-Optik, Friedrich Schiller Universität, Max Wien Platz 1, 07743 Jena, Germany; friedhelm.bechstedt@uni-jena.de

**Keywords:** B24N24, nanocapsules, density functional theory, time-dependent density functional theory, electronic properties, vibrational properties, magnesium batteries

## Abstract

We report on DFT-TDDFT studies of the structural, electronic and vibrational properties of $B_{24}N_{24}$ nanocapsules and the effect of encapsulation of homonuclear diatomic halogens ($Cl_{2}$, $Br_{2}$ and $I_{2}$) and chalcogens ($S_{2}$ and $Se_{2}$) on the interaction of the $B_{24}N_{24}$ nanocapsules with the divalent magnesium cation. In particular, to foretell whether these BN nanostructures could be proper negative electrodes for magnesium-ion batteries, the structural, vibrational and electronic properties, as well as the interaction energy and the cell voltage, which is important for applications, have been computed for each system, highlighting their differences and similarities. The encapsulation of halogen and chalcogen diatomic molecules increases the cell voltage, with an effect enhanced down groups 16 and 17 of the periodic table, leading to better performing anodes and fulfilling a remarkable cell voltage of 3.61 V for the iodine-encapsulated system.

## 1. Introduction

Materials are key components in the sphere of energy production, transformation and storage, and first-principle calculations play a critical role in designing and optimizing new advanced energy materials able to meet the high performance requirements of an efficient and sustainable use of electrical energy [1]. The growing electrified world has been dominated by lithium-ion battery (LIB) technology since the 1990s, when LIBs became the backbone of portable electronics, enabling the convenient storage and utilization of energy [2,3,4]. However, market concerns are rising over lithium resource depletion, which will cause the price of lithium to skyrocket with the escalating implementation of LIBs in the automotive industry and the risk of limiting the wide-scale adoption of electric vehicles [5,6,7,8]. Therefore, over the years, numerous earth-abundant metals have been investigated in order to replace lithium in batteries, and the consensus prevailing in the scientific community affirms that the trajectory of rechargeable batteries hinges significantly on the exploration and advancement of alternative battery chemistries. The technologies proposed include sodium, potassium, magnesium, calcium, aluminum and zinc as the active material, and among all these elements, magnesium is one of the most promising materials for the replacement of lithium [9,10,11,12,13,14,15,16,17,18].

Magnesium ranks among the decadal elements as the most prevalent element in the Earth’s crust. It is non-toxic and is characterized by non-monopolized extraction processes, complete recyclability, an elevated theoretical specific capacity, substantial volumetric energy density and a low redox potential. This underscores magnesium’s growing significance as both a supplemental and alternate element in the evolution of the next generation of power sources [19,20,21,22,23,24]. In addition to the better energy capacities, magnesium-ion batteries (MIBs), which share the same working principle as LIBs, have numerous other advantages over them [25,26,27]. First of all, magnesium is safer than lithium; it does not form toxic compounds, and thus manufacturing MIBs would be more efficient and environmentally friendly. Despite the extensive research over the last decade, MIB technology has yet to be in the initial phase, and this is primarily attributable to the evidence that the active materials conventionally suitable for LIBs are deemed merely satisfactory for MIBs [28,29,30]. Overcoming these challenges requires multidisciplinary efforts to develop novel electrodes and electrolytes that can efficiently store and release magnesium ions during charging and discharging cycles [31,32,33]. The successful adoption of this technology could revolutionize the energy storage landscape, offering superior energy density, augmented safety and a diminished environmental impact [34].

The optimal anode materials for MIBs should exhibit several key characteristics. These include the reversible retention of significant quantities of $Mg^{2+}$ ions, environmental friendliness, cost-effectiveness and high electronic as well as ionic conductivity. Additionally, they should refrain from undergoing chemical reactions or dissolution in the electrolyte. Nanomaterials play a pivotal role in advancing ion battery technologies due to their proven efficacy as electrode candidates, offering superior ionic and electronic conductivity when compared with their bulk counterparts [35,36,37,38,39,40,41,42,43,44,45,46,47,48,49]. The synergy between materials science and high-performance computing (HPC) has opened exciting opportunities for accelerating the discovery of novel electrode nanomaterials. In fact, by leveraging HPC capabilities, researchers can employ sophisticated quantum mechanical calculations (e.g., DFT calculations) and molecular dynamics simulations to explore the properties of thousands of potential nanoelectrodes and tackle complex material design challenges [50,51,52]. This computational approach not only accelerates the research and development process but also significantly reduces the costs associated with experimental synthesis and testing, guiding experimental efforts toward the most promising candidates and thus accelerating the overall material discovery timeline.

Among nanomaterials, BN nanostructures (including nanosheets, nanotubes and nanocages, etc.) have sprung up as promising electrode materials thanks to their unique characteristics, exhibiting chemical and structural stability (suitable for use in corrosive environments), low toxicity, high thermal conductivity and excellent mechanical properties (ideal for better control of charge transport within the energy storage system) [53,54,55,56,57]. The characteristics of diverse nanostructured BN systems vary, contingent upon factors such as the synthesis technique, size, diameter and material shape. Notably, the mixed covalent-ionic B-N bond exerts a substantial influence on the structure of BN allotropes, affording the capability to form $sp^{2}$ or $sp^{3}$ linkage structures. Generally, BN is unsuitable for deployment as an anode electrode in rechargeable batteries due to its wide band gap energy range of 4–6 eV, rendering it an insulator. Additionally, the B-N π bond exhibits limited movement, given the low degree of electron pair delocalization, leading to a scarcity of free electrons in BN [58,59,60]. Nevertheless, a diverse array of methodologies and approaches, encompassing vacancy creation, doping, defect engineering, composite formation, and judicious chemical functionalization, may be employed to proficiently modulate the bandgap (facilitating conductivity) and enhance both electron conductivity and cation–π interaction on the BN surface. The latter is particularly critical for bolstering the alkali-ion storage capacity in a material [61,62,63,64].

Specifically, BN fullerene-like nanostructures have garnered attention since the fullerene’s discovery [65]. The smallest nanocage, $B_{12}N_{12}$, featuring tetragonal BN rings isolated by hexagonal BN rings, has become noteworthy following its synthesis by Oku et al. [66,67,68,69,70,71,72,73]. The potential application of $B_{12}N_{12}$ nanocages as anode materials has been suggested for lithium-ion [74], sodium-ion [75] and magnesium-ion batteries [76,77]. Given the intriguing physical and chemical properties of $B_{12}N_{12}$, the preparation and characterization of fullerene dimers, such as $B_{24}N_{24}$, have garnered substantial attention both experimentally and theoretically. Wu et al. [78] identified the tubular form of $B_{24}N_{24}$ as the most stable structure, resulting from the connection of two six-membered rings of $B_{12}N_{12}$. In this article, systematic calculations in the DFT framework are used to explore $B_{24}N_{24}$ nanocapsules and the interaction of magnesium and magnesium ions with $B_{24}N_{24}$ endonanocapsules (Endon), particularly nanocapsules encapsulating homonuclear diatomic halogen and chalcogen molecules, to uncover if these systems are promising anodes for MIBs. This paper is organized as follows. In Section 2, we pose the computational methods and theoretical approaches employed to characterize these anodes. The formation energies, the interaction energies, the Raman and optical absorption spectra as well as the cell voltages are reported and examined in detail in Section 3 for the endonanocapsules. Lastly, our conclusions are gathered in Section 4.

## 2. Materials and Methods

### 2.1. Geometries and Energies

In the present contribution, outcomes derived from first-principles calculations within the density functional theory framework are found using the DMOL3 package [79,80,81] within Materials Studio [82]. The calculations incorporated the generalized gradient approximation with Perdew–Burke–Ernzerhof exchange-correlation functional [83,84,85] and Grimme’s DFT-D dispersion correction to account for van der Waals interactions [86,87,88]. Electronic wavefunctions were expanded using atom-centered basis functions defined on a dense numerical grid. The chosen basis set, double numerical plus polarization (DNP), was subject to a global cut-off radius of 4.5 Å. This cut-off value ensured atomic energies with an accuracy of 0.1 eV/atom, allowing for calculations without a significant loss of precision. Geometry optimizations were carried out by employing a scheme based on delocalized internal coordinates, and the convergence thresholds for the energy change, maximum force and maximum displacement during geometry optimization were set to $10^{-5}$ Hartree, 0.002 Hartree/Å and 0.001 Å, respectively.

### 2.2. Electrochemical Reactions and Thermodynamics

The estimation of the interaction energy between Mg and Mg^2+^ with the designated endonanocapsule was conducted to assess the comparative efficacy of adsorption. This evaluation was performed using the following equation:

(1) $$E_{interaction}=E_{Endon@Mg^{0/2+}}-E_{Endon}-E_{Mg^{0/2+}}$$

where $E_{Endon@Mg^{0/2+}}$ are the total energies of the endonanocapsules with Mg or with Mg^2+^; $E_{Endon}$ is the energy of the geometry-optimized isolated endonanocapsule and $E_{Mg^{0/2+}}$ is the total energy of the magnesium or magnesium ion. Considering the application of $B_{24}N_{24}$ endonanocapsules as anodes in magnesium-ion batteries, the electrochemical reactions at the anode and cathode can be simplified as follows:

(2) $$\begin{array}{c} Endon@Mg⟶Endon@{Mg}^{2+}+2e^{-}\\ {Mg}^{2+}+2e^{-}⟶Mg \end{array}$$

Therefore, the global reaction in the magnesium-ion battery cell will be

(3) $$Endon@Mg+{Mg}^{2+}⟶Endon@{Mg}^{2+}+Mg+\Delta G_{cell}$$

where $\Delta G_{cell}$ is the Gibbs free energy change of the cell’s total reaction. For the theoretical assessment of the average open circuit intercalation potential ($V_{cell}$) in this cell, the Nernst equation was employed:

(4) $$\Delta G_{cell}=-zFV_{cell}$$

where F is the Faraday constant (96,500 C/mol) and *z* = +2 represents the charge of the divalent magnesium ion, which acts as a working cation in the electrolyte. The $\Delta G_{cell}$ term was assessed initially while neglecting volume and entropy variations and solely taking into account the variation ΔE of the total energy, where $\Delta G_{cell}\approx \Delta E$. Therefore, the $V_{cell}$ of the reaction considered above can be derived from the computed total energies as follows:

(5) $$V_{cell}=\frac{\left(E_{Endon@Mg}-E_{Mg}\right)-\left(E_{Endon@{Mg}^{2+}}-E_{{Mg}^{2+}}\right)}{2F}.$$

As deducible from the aforementioned equation, a heightened voltage ($V_{cell}$) is anticipated for $B_{24}N_{24}$ endonanocapsules, in which the interaction with the magnesium ion surpasses that with magnesium. Such systems may potentially serve as high-performance anode materials for magnesium-ion batteries. Afterward, to account for entropy effects, the Gibbs free energy change of the cell reaction, as described by Equation (3), was computed based on the outcomes derived from frequency analysis at 298.15 K and 1 atm of pressure:

(6) $$\Delta G_{cell}=G_{Mg}+G_{Endon@{Mg}^{2+}}-G_{{Mg}^{2+}}-G_{Endon@Mg}$$

where G is the zero-point energy-corrected Gibbs free energy of the investigated system [89]. The voltage $V_{cell}$ was then calculated using the Nernst equation (Equation (4)). The formation energy for the B_24_N_24_ nanocage and that for the endonanocapsules are each defined as follows:

(7) $$E_{f}=\left(E_{B_{24}N_{24}}-24\varepsilon_{B(s)}-24\varepsilon_{N_{2}\left(g\right)}\right)/48$$

(8) $$E_{f}=\left(E_{Endon}-24\varepsilon_{B(s)}-24\varepsilon_{N_{2}\left(g\right)}-2\varepsilon_{X_{2}}\right)/50$$

where $E_{B_{24}N_{24}}$ and $E_{Endon}$ are the total energies of the primeval nanocage and of each endonanocapsule, respectively, whereas $\varepsilon_{B(s)}$, $\varepsilon_{N_{2}\left(g\right)}$ and $\varepsilon_{X_{2}}$ are the total energies per atom of the trigonal solid boron, N_2_ molecule and the halogen or chalcogen atom, respectively. The sign and value of the formation energies indicate a measure of the stability or instability for each system.

### 2.3. Raman and Optical Absorption Spectra

Raman spectroscopy exploits the Raman effect, involving the inelastic scattering of monochromatic light [90]. This interaction with the vibrations results in a shift in the energy of incident photons. The ensuing spectra are instrumental in investigating vibrational, rotational and other low-frequency modes in a system. The energy shift is dictated by the vibrational frequency, while the fraction of light undergoing inelastic scattering is determined by the spatial derivatives of the macroscopic polarization. In DMOL3, the Raman intensities and activities are computed using the finite differentiation technique. Multiple gradient calculations are executed under varying electric fields to generate the polarizability tensor derivative, which fundamentally defines the Raman activity. At a given incident light frequency and temperature, assuming a plane-polarized incident laser beam, the first-order differential Raman cross-section (intensity) for the Stokes component of the ith eigenmode far from resonance ($\nu_{i}$) [91] is calculated as follows:

(9) $$\frac{d\sigma_{i}}{d\Omega }=\frac{{\left(2\pi \nu_{S}\right)}^{4}}{c^{4}}{\left|{\hat{e}}_{S}\frac{\partial \tilde{\alpha }}{\partial Q_{i}}{\hat{e}}_{L}\right|}^{2}\frac{h\left(n_{i}^{b}+1\right)}{8\pi^{2}\nu_{i}}$$

where ${\hat{e}}_{S}$ and ${\hat{e}}_{L}$ are the unit vectors of the electric-field polarization for the scattered and incident light, respectively, $Q_{i}$ is a normal-mode coordinate, $\nu_{S}$ is the frequency of the scattered light, $\tilde{\alpha }$ is the polarizability tensor and $n_{i}^{b}$ is the Bose–Einstein statistical factor. Given that the frequency of the scattered light $\nu_{S}$ derives from the frequency of the incident light $\nu_{0}$, according to $\nu_{S}=\left(\nu_{0}-\nu_{i}\right)$, this enables the evaluation of Raman intensities under the experimental conditions T = 298.15 K and λ = 514.5 nm (green laser).

The time-dependent density functional theory in the linear response regime is a extremely efficient method for determining excitation energies and optical spectra [92]. Since the external field is small, it is treated as a time-dependent perturbation. Then, the linear response function (the frequency-dependent dynamic polarizability tensor) depends only on the ground state density, and it has poles at the excitation energies of the system, which can be computed by resolving the following eigenvalue problem [93,94]:

(10) $$Q_{ij,kl}F_{I}=\Omega_{I}^{2}F_{I}$$

where $F_{I}$ represents the multi-determinantal excited states, $\Omega_{I}$ represents the excitation energies and $Q_{ij,kl}$ represents the matrix elements, with the labels ik describing the occupied orbitals and jl describing the unoccupied ones. Iterative diagonalization [95,96] is used to calculate the product of $Q_{ij,kl}$ operating on the trial excitation vector F.

## 3. Results and Discussion

### 3.1. $B_{24}N_{24}$ Nanocapsule

**Figure 1:**
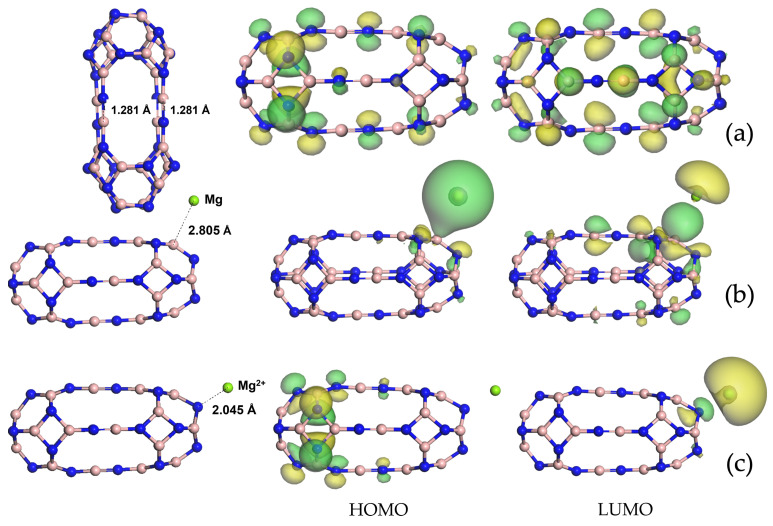
Optimized structures and visualization of frontier molecular orbitals for the $B_{24}N_{24}$ nanocapsule (**a**), $B_{24}N_{24}$@Mg (**b**) and $B_{24}N_{24}$@Mg^2+^ (**c**). Nitrogen atoms are denoted in blue, boron atoms in pink and magnesium atoms in green. Distances are in Å.

The $B_{24}N_{24}$ nanocapsule was constructed by arranging 24 boron atoms and 24 nitrogen atoms in a specific pattern, resulting in an optimized hollow cage-like structure. At the GGA-PBE level of theory, the dimerization of $B_{12}N_{12}$ is observed through the breaking of four B$N_{6-6}$ (hexagonal rings close to hexagonal rings) single bonds and the consequent formation of four B=N bridges, all of which are 1.281 Å long. The stability of the dimers is confirmed by a negative formation energy $E_{f}$ = −0.48 eV/atom (see Table 1). At the baseline, the adsorption of Mg and magnesium ions at various initial sites was investigated, and the total energy minimum was found for the Mg adsorption, preferentially on B (see Figure 1b), as well as that for the Mg^2+^ adsorption on N (see Figure 1c). The distance between the B_24_N_24_ nanocapsule and the elemental Mg was 2.805 Å, whereas a decrease was observed in this quantity to 2.045 Å in the case of the cation Mg^2+^. This tendency was reflected in the interaction energy value (see Table 1), which was negative (−6.50 eV) in the case of Mg^2+^ adsorption and positive (+0.27 eV) in the case of Mg, and it was outlined by a bigger Lewis acid–base interaction regarding the ion.

Also, in terms of electronic properties, the effect of the Mg and Mg^2+^ adsorption was completely distinct, leading to an energy gap of 2.369 eV for the B_24_N_24_@Mg and 0.285 eV for the B_24_N_24_@Mg^2+^. When considering the B_24_N_24_ nanocapsule as anode material for MIBs, the cell voltage calculated with Equation (4) was 3.38 V, which is much bigger than the 2.7 V found in the literature for the B_12_N_12_ nanocage [76,77]. From Equation (4), it is evident that a weaker Mg–B_24_N_24_ interaction combined with a stronger Mg^2+^–B_24_N_24_ interaction is conducive to an increased cell voltage, a quantity of fundamental importance for rechargeable batteries. Additionally, the hollow structure of the B_24_N_24_ system provides an excellent environment for encapsulating other molecules or nanoparticles.This fact opens the possibility of encapsulating B_24_N_24_ with homonuclear diatomic molecules (see Figure S1 in the Supplementary Materials) and examine whether a better Mg^2+^–B_24_N_24_ interaction can be obtained in the case of endonanocapsules.

### 3.2. Diatomic Halogen Endonanocapsules

**Figure 2:**
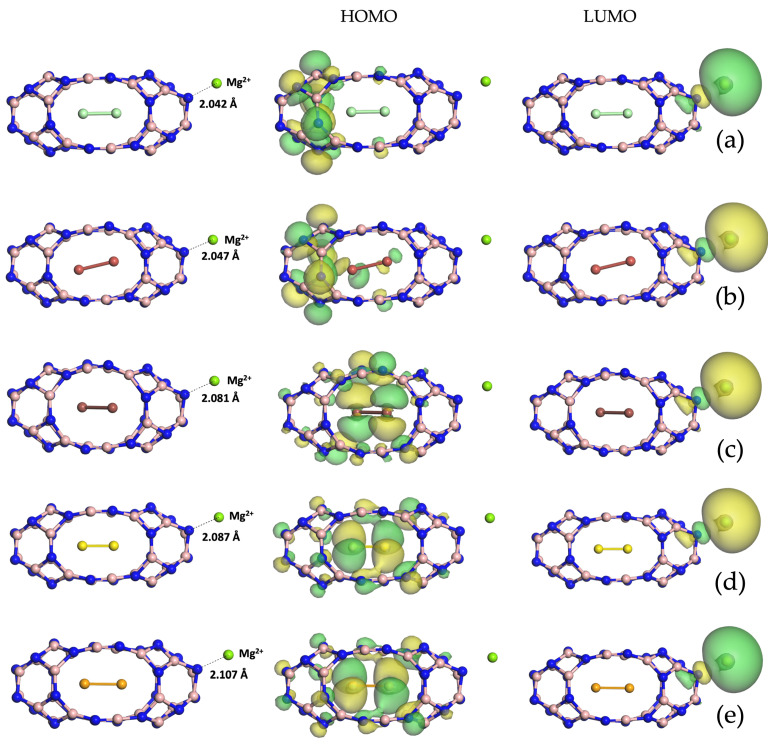
Optimized structures and visualizations of frontier molecular orbitals for (from top to bottom) $Cl_{2}$/$B_{24}N_{24}$@$Mg^{2+}\left(a\right)$, Br_2_/B_24_N_24_@Mg^2+^ (**b**), $I_{2}$/$B_{24}N_{24}$@$Mg^{2+}\left(c\right)$, S_2_/B_24_N_24_@Mg^2+^ (**d**) and $Se_{2}$/$B_{24}N_{24}$@$Mg^{2+}\left(e\right)$.

**Figure 3:**
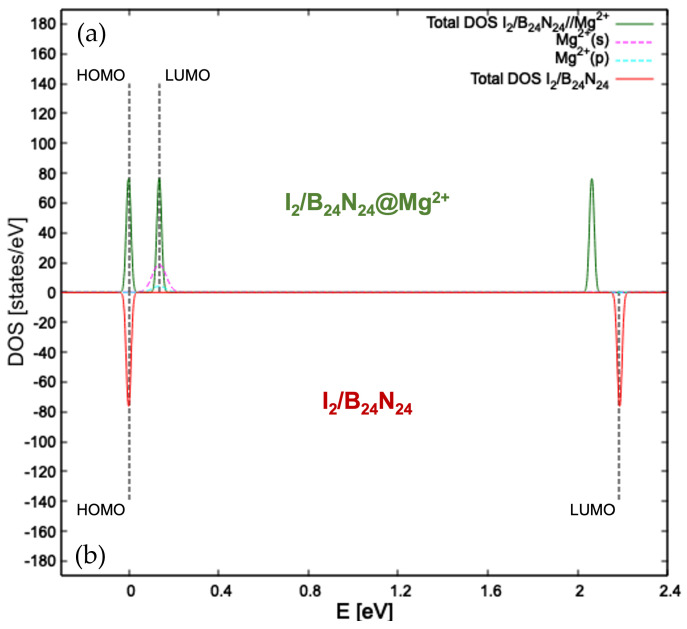
Details of the atom- and angular momentum-projected DOS for $I_{2}$/$B_{24}N_{24}$@Mg^2+^ (**a**) and the total density of states computed for $I_{2}$/$B_{24}N_{24}$ (**b**) at the GGA-PBE level of theory. The HOMO is taken to be zero for the energy scale. Dashed black lines indicate the HOMO and LUMO.

Diatomic halogen encapsulation entails an enlargement of the $B_{24}N_{24}$ nanocapsules, as visible in the relaxed structures in Figure S1 in the Supplementary Material, with an elongation of the B=N bridges. Their lengths increased when going from chlorine to iodine and became 1.289 Å for $Cl_{2}$/$B_{24}N_{24}$, 1.301 Å for $Br_{2}$/$B_{24}N_{24}$ and 1.311 Å for $I_{2}$/$B_{24}N_{24}$. The same trend can be seen for the energy gap value (see Table 1), which was 1.473 eV for the system enclosing $Cl_{2}$, 1.671 eV for the one with $Br_{2}$ and 2.184 eV for the one with $I_{2}$. The minimum total energy for the adsorption of both magnesium and magnesium ion onto $Cl_{2}$/$B_{24}N_{24}$ (see Figure 2a), $Br_{2}$/$B_{24}N_{24}$ (see Figure 2b) and $I_{2}$/$B_{24}N_{24}$ (see Figure 2c) fell into its adsorption on N. The frontier molecular orbitals plotted for the three endonanocapsules show how the HOMO arose from the hybridization of p orbitals of N and B for $Cl_{2}$/$B_{24}N_{24}$@$Mg^{2+}$ and $Br_{2}$/$B_{24}N_{24}$@$Mg^{2+}$, while for $I_{2}$/$B_{24}N_{24}$@Mg^2+^, it arose from the hybridization of p orbitals of N, B and those of the encapsulated diatomic iodine, as confirmed by the pDOS in Figure 3a. The LUMO retained the spherical shape of the s orbital of Mg^2+^ for all the three systems. The Mg distance from the endonanocapsules was 2.088 Å, 2.145 Å and 3.254 Å, while the Mg^2+^ was located at 2.042 Å, 2.046 Å and 2.081 Å from the $Cl_{2}$/$B_{24}N_{24}$@Mg^2+^, Br_2_/B_24_N_24_@Mg^2+^ and $I_{2}$/$B_{24}N_{24}$@Mg^2+^ structures, respectively.

The increase in the distance between Mg^2+^ and the halogen endonanocapsules was due to a more positive global Mulliken charge on the endonanocapsule, which was 1.007 for Cl_2_/B_24_N_24_@Mg^2+^, 1.017 for Br_2_/B_24_N_24_@Mg^2+^ or 1.122 for I_2_/B_24_N_24_@Mg^2+^, with these values obtained with a progressively more negative Mulliken charge on the encapsulated diatomic halogens. Moreover, it was found that a more negative Mulliken charge on the enclosed molecule had two major effects: (1) boosting the interaction of the halogen endo- nanocapsule with Mg^2+^, as evident from the interaction energy values (see Table 1), which were −6.58 eV for Cl2/B_24_N_24_@Mg^2+^ in comparison with −7.24 eV for I_2_/B_24_N_24_@Mg^2+^, and (2) diminishing the HOMO-LUMO energy gap at the PBE-GGA level to 0.289 eV for Cl_2_/B_24_N_24_@Mg^2+^ and 0.136 eV for I_2_/B_24_N_24_@Mg^2+^. The insulator HOMO-LUMO gap of B_24_N_24_ (4.261 eV) was thus reduced to improve conductivity.

**Figure 4:**
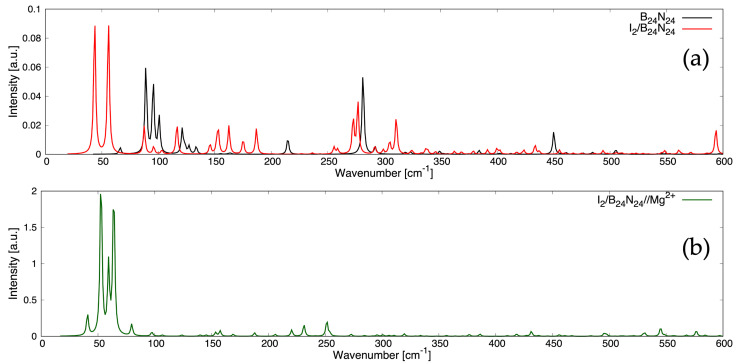
Raman spectra of $B_{24}N_{24}$ (**a**), $I_{2}$/$B_{24}N_{24}$ (**a**) and $I_{2}$/$B_{24}N_{24}$@$Mg^{2+}$ (**b**) at T = 298.15 K and at an excitation wavelength λ = 514.5 nm.

To discern the nature of the chemical bonds and the subtleties in the atomic arrangements as well as predict the measurable features, Raman spectra were computed for B_24_N_24_ (see Figure 4a), I_2_/B_24_N_24_ (see Figure 4a) and I_2_/B_24_N_24_@Mg^2+^ (see Figure 4b). Raman shifts reveal a wealth of information about the molecular vibrations, which rule the ther- mal and electrical conductivity, thermal expansion and mechanical properties. Breathing and deformation modes of the B-N π bonds appeared at 89.02 cm^−1^, 95.46 cm^−1^ and 281.25 cm^−1^ for B_24_N_24_, and at 116.51 cm^−1^, 162.52 cm^−1^ and 276.69 cm^−1^ for I_2_/B_24_N_24_ (see Tables S1 and S2 in the Supplementary Materials), since these vibrations involved light atoms and strong bonds, while two librational modes at 43.64 cm^−1^ and 87.49 cm^−1^ and one translational mode at 55.78 cm^−1^ of the encapsulated I_2_ occurred at a lower Raman shift for I_2_/B_24_N_24_ since these vibrations involved heavier atoms (see Figure 4a). Furthermore, with regard to I_2_/B_24_N_24_@Mg^2+^, the analysis of the intensities and positions of the Raman peaks unveiled rocking modes associated with the Mg^2+^ adsorbed onto the endonanocapsule at 52.66 cm^−1^, 59.31 cm^−1^ and 63.94 cm^−1^, while two librations of I_2_ occurred at 40.81 cm^−1^ and 79.69 cm^−1^, and one translation was observed at 97.45 cm^−1^ (see Figure 4b). The Mg^2+^ adsorption drastically changed the Raman spectrum for shifts above 80 cm^−1^. The intense Raman peaks in the ranges of 80–190 cm^−1^ and 260–320 cm^−1^ still appeared in the spectrum of I_2_/B_24_N_24_@Mg^2+^ (see Figure 4b), even though they were barely visible compared with the more pronounced peaks associated with the modes of the magnesium ion.

**Figure 5:**
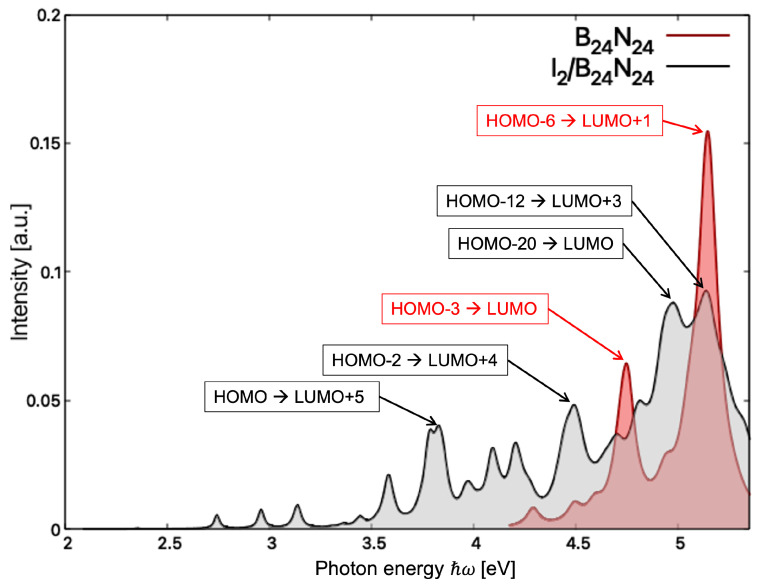
Optical absorption spectra of $B_{24}N_{24}$ (red) and $I_{2}$/$B_{24}N_{24}$ (gray), calculated within TDDFT with the ALDA-XC kernel. Transitions related to relevant peaks are shown.

The optical absorption spectra in TDDFT for B_24_N_24_ and I_2_/B_24_N_24_ at the ALDA-XC Kernel level of theory exhibited peaks above 4 eV and 2 eV, respectively (see Figure 5). The lowest energy peak arose from the weak HOMO-LUMO electronic excitation in I_2_/B_24_N_24_ at 2.35 eV, while the same excitation in B_24_N_24_ occurred at 4.29 eV. For B_24_N_24_, two rather intense peaks were observed, with one at 4.75 eV which corresponded to the excitation involving the p-states of N in the B-N π bonds (HOMO-3) and the LUMO and the other one, the highest energy peak, corresponding to the strong excitation between the p states of N (HOMO-6) and the p states of B (LUMO+1) in B_24_N_24_ at 5.14 eV. For I_2_/B_24_N_24_, four relevant peaks are visible in Figure 5: one at 3.83 eV involving the HOMO and the p states of B (LUMO+5), one at 4.50 eV involving the p states of I_2_ (HOMO-2) and the p states of B (LUMO+4), one at 5.06 eV corresponding to the electronic excitation between the p states of N (HOMO-20) and the p states of I_2_ (LUMO) and the last at 5.14 eV corresponding to the excitation between the I_2_ bond (HOMO-12) and the p states of B (LUMO+3). A detailed analysis of the positions of the absorption peaks in TDDFT and DFT is summarized in Tables S4 and S5 in the Supplementary Materials. In any case, the comparison of the absorption edges of B_24_N_24_ and I_2_/B_24_N_24_ shows that the absorption spectroscopy can be applied to discriminate the nanocapsules from the endonanocapsules.

**Figure 6:**
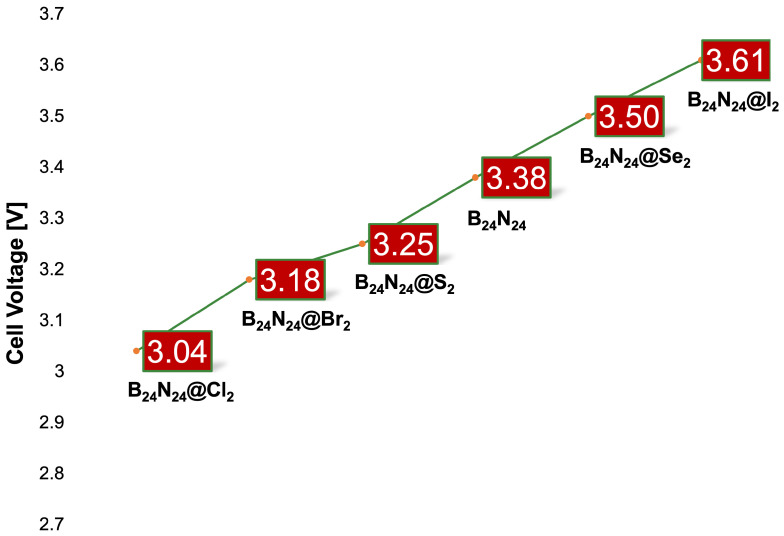
Voltage $V_{cell}$ trend at 298.15 K and 1 atm for the examined nanostructures.

The atom- and orbital-projected density of states of I_2_/B_24_N_24_ (see Figure 3b) show that HOMO and LUMO were separated well, resulting in a band gap of 2.184 eV. On the other side, the pDOS for I_2_/B_24_N_24_@Mg^2+^ (see Figure 3a) exhibited the frontier orbitals to be quite close in terms of energy, with the s orbital of the magnesium ion deeply contributing to the LUMO. Its spherical shape is visible in Figure 2a–e, from which the halogen and chalcogen molecules are independently encapsulated. Consequently, a further redshift of the absorption edge is expected after Mg^2+^ adsorption. These facts suggest the idea, confirmed by the Mulliken population analysis, that diatomic halogen encapsulation leads to a cell voltage better than that of B_24_N_24_. In fact, calculations performed at 1 atm and 298.15 K (see Equation (5)) for the I_2_ endonanocapsule obtained a cell voltage of 3.61 V, exceeding the 3.38 V of the pristine nanocapsule (see Figure 6).

### 3.3. Diatomic Chalcogen Endonanocapsules

The geometrically optimized structures of diatomic chalcogens encapsulating B_24_N_24_ (see Figure S1) display how the B=N bonds lengthened with an increase in the atomic number of the chalcogen atom, changing from 1.289 Å for the S_2_/B_24_N_24_ to 1.292 Å for the Se_2_/B_24_N_24_ in comparison with 1.281 Å for the pristine B_24_N_24_. The trend of the energy gaps was the same as that observed for the halogen endonanocapsules, even though the values were significantly smaller (see Table 1), being 0.004 eV for the S_2_ endonanocapsule and 0.037 eV for the Se_2_ endonanocapsule. The total energy minima for Mg and Mg^2+^ adsorption on S_2_/B_24_N_24_ (see Figure 2d) and S_2_/B_24_N_24_ (see Figure 2e) correspond to a position near an N atom. The frontier molecular orbitals plotted for the two endonanocapsules show how the HOMO arose from the hybridization of the p orbitals of N, B and those of the encapsulated diatomic chalcogens for S_2_/B_24_N_24_@Mg^2+^ and Se_2_/B_24_N_24_@Mg^2+^, highlighting the charge transfer from the encapsulated species to the B_24_N_24_ surface. The LUMO retained the spherical shape of the s orbital of Mg^2+^ for both systems. It can be observed that both the Mg and Mg^2+^ distances were comparable with those found for the chalcogen endonanocapsule. In fact, Mg was located at 2.107 Å and 2.140 Å from Se_2_/B_24_N_24_ and Se_2_/B_24_N_24_, respectively, while Mg^2+^ was within 2.087 Å from the nanocapsule enclosing S_2_ and 2.107 Å from the one enclosing Se_2_. In the case of the chalcogen endonanocapsule, Mg^2+^ was even more distant, in agreement with a global Mulliken charge on the enonancapsule, which was more positive than the cases seen above (1.111 elementary charges for Se_2_/B_24_N_24_ and 1.154 for Se_2_/B_24_N_24_). The local Mulliken charge on the chalcogen molecule was negative, and as stated above, it was related to an improvement in the interaction energy with Mg^2+^, which was −6.94 eV for S_2_ encapsulation and −7.43 eV for Se_2_ encapsulation, as well as a decrease in the PBE-GGA energy gap, which was 0.120 eV for S_2_/B_24_N_24_@Mg^2+^ and 0.086 eV for Se_2_/B_24_N_24_@Mg^2+^ (see Table 1). The cell voltage calculations carried out at 1 atm and 298.15 K (see Equation (5)) returned 3.25 V and 3.50 V for the S_2_ and Se_2_ endonanocapsules, respectively. Despite the encapsulation, S_2_/B_24_N_24_ was less efficient than the pristine nanocapsule as an anode for magnesium batteries. Instead, Se_2_/B_24_N_24_ was undoubtedly capable of reaching a voltage higher than that of B_24_N_24_ but still lower than that observed for I_2_/B_24_N_24_.

The values of the open circuit potentials calculated in this DFT-TDDFT study for diatomic halogen and chalcogen B_24_N_24_ endonanocapsules are indeed significant in comparison with those obtained for other low-dimensional materials explored as anodes for MIBs via first principles calculations. In fact, earlier studies reported values for the open circuit potential such as ∼1.6 V for defective graphene [97], 0.89 V for a single layer of phosphorene [98] and 0.85 V for a single layer of WS_2_ [99].

## 4. Conclusions

The atomic arrangement of the B_24_N_24_ nanocapsule was highly symmetrical and stable, allowing it to retain its shape and structural integrity, which is essential for its potential applications in various fields, namely catalysis and energy storage inter alia. Moreover, its particular geometrical structure enables encapsulation with molecules, possibility being exploitable for enhancing the electrochemical properties of this nanomaterial.

Based on the DFT-TDDFT approach, this study investigated the effect of the encapsulation of homonuclear diatomic halogens and chalcogens and the interaction of the resulting endonanocapsules with the divalent magnesium cation. Indeed, we demonstrated that a stronger interaction with Mg^2+^ eads to an increased cell voltage, with a positive impact on the performance of rechargeable magnesium batteries.

The Mulliken population analysis made clear that a more negative charge on the enclosed molecule has three important consequences: boosting the interaction of the halogen or chalcogen endonanocapsule with Mg^2+^, evident from the interaction energy values; diminishing the HOMO-LUMO energy gap at the PBE-GGA level of theory and obtaining a cell voltage better than that of the pristine B_24_N_24_. Given that I_2_/B_24_N_24_ turned out to be capable of reaching the highest cell voltage among the studied systems, an in-depth investigation of this endonanocapsule was performed, including the calculation of the Raman and optical adsorption spectra.

The Raman spectra were enabled to identify the main vibrations of the B-N π bonds, those of the encapsulated I_2_ molecule and those of the adsorbed magnesium ion. In parallel, the optical absorption spectra highlighted the characteristic electronic excitations of I_2_/B_24_N_24_, making a comparison with those of B_24_N_24_. While the analysis of the vibrational and optical properties shed light upon the nature of chemical bonds and the subtleties in atomic arrangements on one hand, on the other hand, it could drive the experimental characterization of these nanomaterials with both in situ and operando spectroscopy.

At least thus far, our studies of the geometry, the adsorption site, the interaction strength and the resulting cell voltage of the magnesium ion adsorption on B_24_N_24_ encapsulating a diatomic halogen or chalcogen molecule pave the way toward experimental preparation as anodes for magnesium-ion batteries. Moreover, the properties of these nanomaterials showed advanced performance, with a low environmental impact arising out of the natural abundance of Mg and I and the chemical inertness of BN.

In summary, our investigation of B_24_N_24_ encaspulation with homonuclear diatomic halogens and chalcogens, as well as the interaction of the resulting endonanocapsules with homonuclear diatomic halogens and chalcogens, as well as the interaction of the resulting endonanocapsules with Mg^2+^ cations, clearly indicates that a stronger interaction with these ions leads to an increased cell voltage. Consequently, it enhances their performance as negative electrodes for rechargeable magnesium batteries. In the case of the iodine-encapsulated anode, a remarkable cell voltage of 3.61 V was reached.

## Figures and Tables

**Table 1:** Interaction energies ($E_{inter}$), HOMO-LUMO energy gap ($E_{gap}$), cell voltage at 298.15 K and 1 atm ($V_{cell}$), cell Gibbs free energy change ($\Delta G_{cell}$) and formation energies ($E_{f}$) for the nanostructures investigated.

Capsule	$E_{inter}$ (eV)	$E_{gap}$ (eV)	$V_{cell}$ (V)	$\Delta G_{cell}$ (kcal/mol)	$E_{f}$ (eV/atom)
$B_{24}N_{24}$	-	4.261	-	−156.1	−0.48
$B_{24}N_{24}$@Mg	+0.27	2.369	-	-	-
$B_{24}N_{24}$@$Mg^{2+}$	−6.50	0.285	3.38	-	-
$Cl_{2}$ **/** $B_{24}N_{24}$	-	1.473	-	−140.2	−0.40
$Cl_{2}$/$B_{24}N_{24}$@Mg	−0.41	0.067	-	-	-
$Cl_{2}$/$B_{24}N_{24}$@$Mg^{2+}$	−6.58	0.289	3.04	-	-
$Br_{2}$ **/** $B_{24}N_{24}$	-	1.671	-	−146.7	−0.37
$Br_{2}$/$B_{24}N_{24}$@Mg	−0.19	0.169	-	-	-
$Br_{2}$/$B_{24}N_{24}$@$Mg^{2+}$	−6.58	0.360	3.18	-	-
$I_{2}$ **/** $B_{24}N_{24}$	-	2.184	-	−166.7	−0.29
$I_{2}$/$B_{24}N_{24}$@Mg	+0.11	0.851	-	-	-
$I_{2}$/$B_{24}N_{24}$@$Mg^{2+}$	−7.24	0.136	3.61	-	-
**$S_{2}$/$B_{24}N_{24}$**	-	0.004	-	−150.0	−0.42
$S_{2}$/$B_{24}N_{24}$@Mg	−0.49	0.081	-	-	-
$S_{2}$/$B_{24}N_{24}$@$Mg^{2+}$	−6.94	0.120	3.25	-	-
$Se_{2}$ **/** $B_{24}N_{24}$	-	0.037	-	−161.6	−0.38
$Se_{2}$/$B_{24}N_{24}$@Mg	−0.43	2.272	-	-	-
$Se_{2}$/$B_{24}N_{24}$@$Mg^{2+}$	−7.43	0.086	3.50	-	-

## Data Availability

Dataset available on request from the authors.

## Supplementary Materials

The following supporting information can be downloaded at: https://www.mdpi.com/article/10.3390/nano14030271/s1, Figure S1: Geometrically optimized structures and molecular orbitals plots for endonanocapsules; Figure S2: Geometrically optimized structures and molecular orbitals plots for endonanocapsules adsorbing Mg; Figure S3: Energy level diagram for diatomic iodine endonanocapsule; Table S1: Vibrational analysis of the BN nanocapsule; Table S2: Vibrational analysis of the diatomic iodine endonanocapsule; Table S3: Vibrational analysis of the diatomic iodine endonanocapsule adsorbing Mg-ion; Table S4: Optical absorption analysis of the BN nanocapsule; Table S5: Optical absorption analysis of the diatomic iodine endonanocapsule.

## Abbreviations

The following abbreviations are used in this manuscript:

ALDA	Adiabatic local density approximation
BN	Boron nitride
DFT	Density functional theory
DFT-D	Dispersion-correction density functional theory
DOS	Density of states
GGA	Generalized gradient approximation
HOMO	Highest occupied molecular orbital
LIBs	Lithium-ion batteries
LUMO	Lowest unoccupied molecular orbital
MIBs	Magnesium-ion batteries
PBE	Perdew–Burke–Ernzerhof exchange-correlation functional
TDDFT	Time-dependent density functional theory
XC	Exchange-correlation
