# Supplementary material for: Structural, Electronic and Vibrational Properties of B24N24 Nanocapsules: Novel Anodes for Magnesium Batteries
Source: Nanomaterials (Basel). 2024 Jan 26;14(3):271. doi: 10.3390/nano14030271 (PMC10856419; doi:10.3390/nano14030271)
Supplement: Supplementary file 1 [file nanomaterials-14-00271-s001.zip › main.pdf]

Supplementary Material
Structural, electronic and vibrational properties of $B_{24}N_{24}$ nanocapsules: novel anodes for magnesium batteries

Domenico Corona,[1] Francesco Buonocore,[2] Friedhelm Bechstedt,[3] Massimo Celino,[2] and Olivia Pulci[1]

[1] *Department of Physics, University of Rome Tor Vergata, and INFN, Via della Ricerca Scientifica 1, 00133 Rome, Italy; domenico.corona@roma2.infn.it (D.C.); olivia.pulci@roma2.infn.it (O.P.)*

[2] *Energy Technologies and Renewable Sources (TERIN) Department, Italian National Agency for New Technologies, Energy and Sustainable Economic Development (ENEA), Casaccia Research Centre, 00123 Rome, Italy; francesco.buonocore@enea.it (F.B.); massimo.celino@enea.it (M.C.)*

[3] *Institut für Festkörpertheorie und -optik, Friedrich Schiller Universität, Max Wien Platz 1, 07743 Jena, Germany; friedhelm.bechstedt@uni-jena.de*

(Dated: January 26, 2024)

I. GEOMETRICAL STRUCTURES

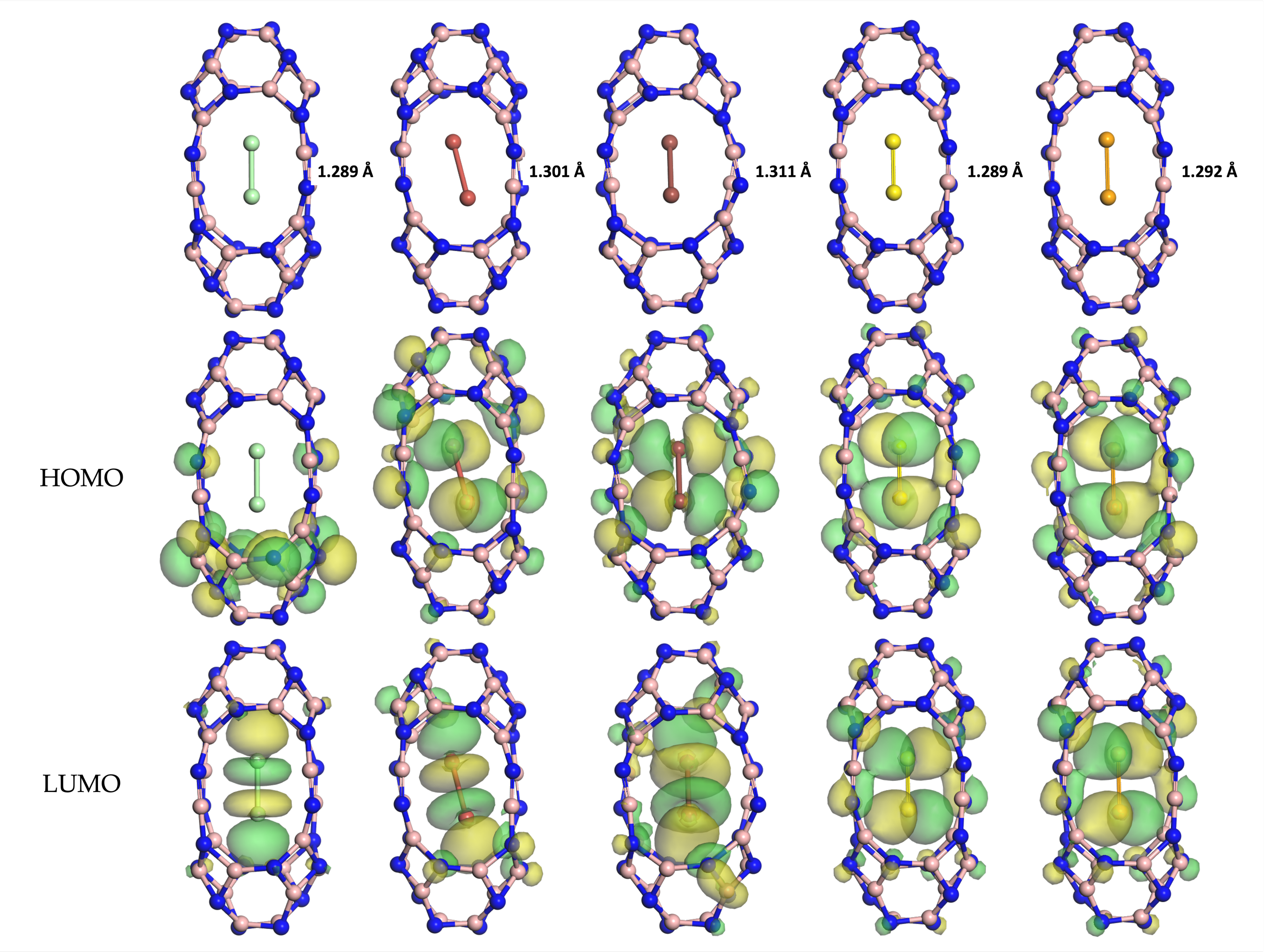

Figure S1. Geometrically optimized structures and molecular orbitals plots for (from left to right): $Cl_2/B_{24}N_{24}$, $Br_2/B_{24}N_{24}$, $I_2/B_{24}N_{24}$, $S_2/B_{24}N_{24}$ and $Se_2/B_{24}N_{24}$. Distances are in Å.

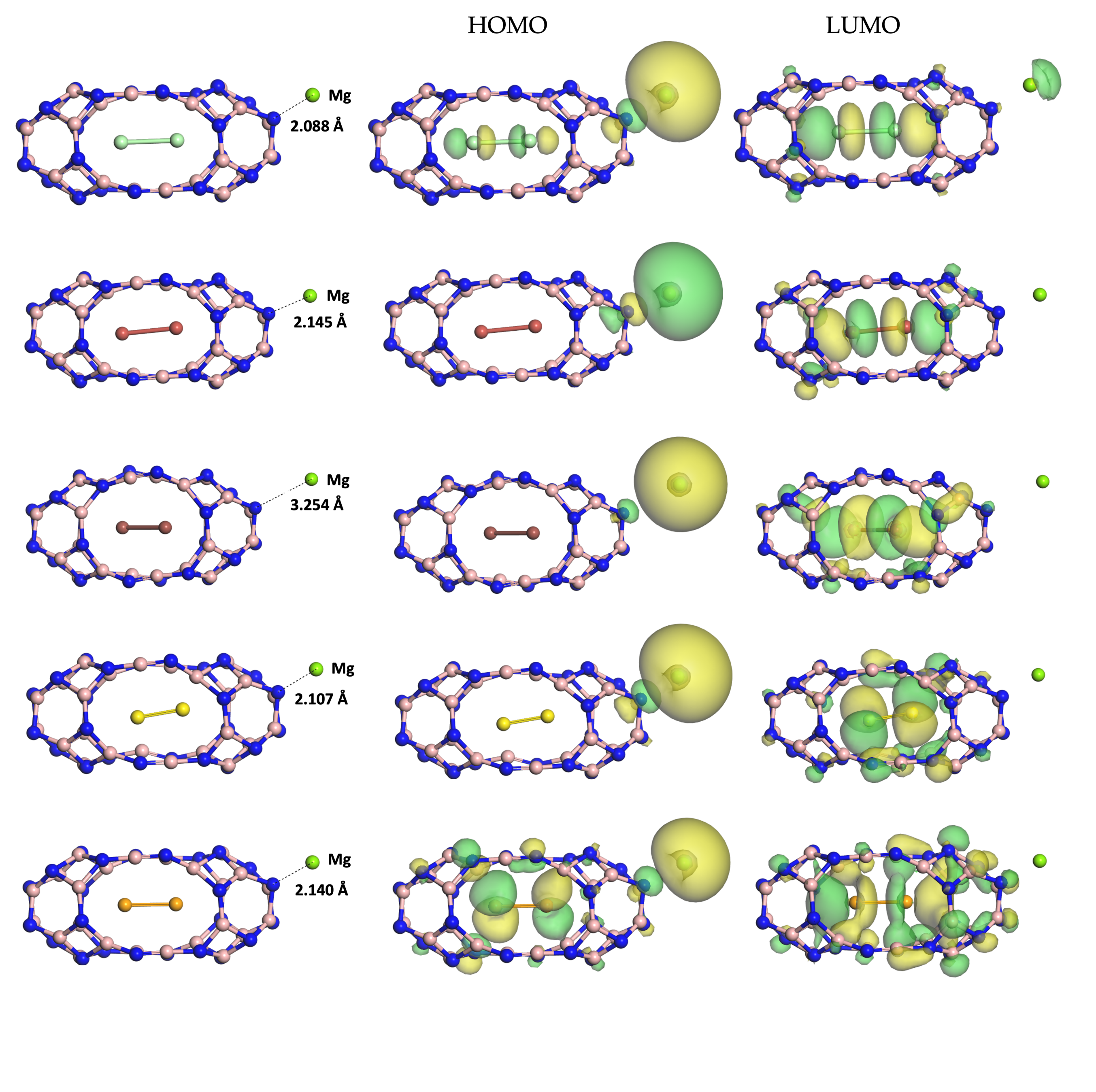

Figure S2. Geometrically optimized structures and molecular orbitals plots for (from top to bottom): $Cl_2/B_{24}N_{24}$@Mg, $Br_2/B_{24}N_{24}$@Mg, $I_2/B_{24}N_{24}$@Mg, $S_2/B_{24}N_{24}$@Mg and $Se_2/B_{24}N_{24}$@Mg. Distances are in Å.

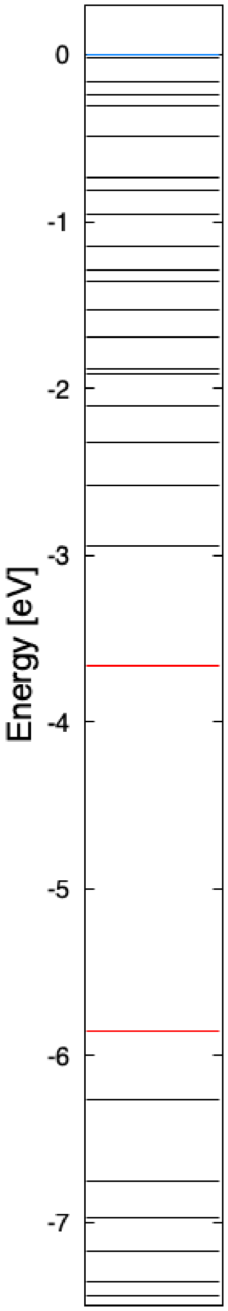

Figure S3. Energy level diagram for $I_2/B_{24}N_{24}$. The energy range considered is between -7.5 eV and the vacuum level (blue line), and the frontier orbitals are denoted in red lines.

II. VIBRATIONAL ANALYSIS

**Frequency (1/cm)**	**Intensity IR (km/mol)**	**Intensity Raman (km/mol)**
66.19	0.01	1.549
89.02	0.78	20.906
95.46	0.75	17.194
100.65	1.16	8.716
121.22	0.06	5.879

123.63	0.31	1.747
127.2	0.04	1.798
133.65	0.14	1.78
155.24	0.07	0.138
162.09	0.83	0.15
163.91	1.8	0.275
171.23	1.53	0.008
214.71	0.22	3.845
234.92	8.69	0.022
243.31	7.31	0.088
277.45	0.03	0.293
281.25	0.02	19.497
292.09	0.03	1.716
318.81	2.18	0.484
323.38	2.02	0.544
334.26	0.26	0.052
337.52	0.54	0.011
349.07	0.92	0.757
352.7	0.15	0.117
356.34	0.15	0.127
373.97	2.41	0.055
374.95	1.81	0.079
384.02	0.02	0.932
388.42	0.08	0.12
401.97	6.2	0.23
404.4	0.42	0.044
412.1	7.66	0.215
450.21	0.07	5.425
461.31	26.65	0.366
475.93	20.58	0.304
482.4	0.41	0.05
484.64	1.48	0.437
491.9	0.48	0.095
504.42	0.41	0.294
505.27	0.3	0.808
510.65	12.86	0.045
512.92	14.17	0.017
514.82	6.65	0.082
541.76	10.46	0.033
542.18	11.02	0.085
546.05	11.98	0.131
549.94	7.72	0.184
552.83	2.4	0.174
565.85	9.94	0.005
571.17	9.35	0.007
577.98	0.14	0.018
582.56	0.75	0.014
588.89	10.21	0.087
596.54	5.23	0.14
602.78	2.89	0.01
606.11	3.48	0.02
628.67	3.82	0.048
634.79	3.69	0.054
635.53	4.91	0.028
665.3	1.33	0.193
668.07	6.13	0.155
685.17	5.55	0.037
690.55	3.91	0.403
691.93	98.07	1.129
695.41	30.03	3.105

698.52	79.66	0.985
717.72	0.05	4.11
724.22	28.34	0.056
728.07	41.6	0.07
728.79	146.78	0.016
729.43	59.26	0.082
733.38	2.02	1.745
735.72	0.72	3.649
740.3	140.04	0.042
742.36	88.23	0.044
766.07	0.41	0.01
772.94	2.12	0.035
785.06	0.71	0.097
785.8	2.62	0.145
792.26	350.95	0.052
794.39	126.06	0.173
799.33	0.75	5.977
835.89	16.73	0.567
839.26	19.09	0.519
855.62	3.86	0.047
861.46	1.63	0.039
863.28	2.76	0.064
863.59	1.84	0.089
866.82	1.27	0.012
881.22	0.17	0.099
881.91	0.71	0.258
882.53	0.53	0.262
887.14	0.13	0.05
906.19	0.36	0.052
912.52	1.18	0.055
958.05	0.36	0.268
976.55	37.15	0.013
978.29	35.19	0.012
1.00E+03	1.08	0.246
1.01E+03	67.49	0.07
1.08E+03	7.27	0.003
1.08E+03	9.14	0.02
1.11E+03	1.28	0.186
1.12E+03	8.76	0.055
1.15E+03	78.31	0.035
1.16E+03	51.27	0.047
1.17E+03	1.81	0.444
1.17E+03	6.1	0.113
1.19E+03	68.16	0.062
1.19E+03	150.72	0.005
1.19E+03	54.99	0.063
1.21E+03	57.63	0.023
1.22E+03	49.76	0.064
1.22E+03	40.03	0.029
1.23E+03	3.01	0.065
1.26E+03	48.48	0.131
1.26E+03	1.65E+03	0.024
1.27E+03	346.67	0.11
1.30E+03	985.72	0.007
1.31E+03	65.93	0.02
1.31E+03	64.04	0.017
1.32E+03	7.85	0.489
1.36E+03	106.03	0.007
1.37E+03	23.43	0.004
1.41E+03	1.94E+03	0.011

1.42E+03	1.81E+03	0.085
1.42E+03	232.15	0.561
1.44E+03	1.35E+03	0.123
1.44E+03	548.32	0.144
1.45E+03	5.37	0.173
1.49E+03	24.24	0.012
1.51E+03	27.25	0.046
1.52E+03	26.44	0.051
1.56E+03	6.13	0.113
1.95E+03	0.31	7.176
1.98E+03	3.61	0.023
1.98E+03	67.74	0.451
2.05E+03	5.72E+03	0.017

Table S1: Vibrational Analysis of the systems $B_{24}N_{24}$.

Frequency (1/cm)	Intensity IR (km/mol)	Intensity Raman (km/mol)
43.64	1.54	32.591
55.78	0.94	31.931
87.49	0.34	6.495
95.91	0.14	1.747
103.24	0.31	0.91
116.51	6.32	7.21
145.77	0.72	2.356
151.67	2.66	1.813
152.97	3.56	5.533
162.52	2.42	6.599
175.08	4.85	3.447
186.94	6.66	6.005
200.78	6.4	0.067
226.76	0.8	0.124
236.43	0.43	0.305
255.6	7.22	1.651
258.83	11.66	1.161
272.52	24.25	8.532
276.69	18.6	12.416
291.74	12.5	1.877
299.21	12.46	0.961
303.85	2.61	0.326
304.91	1.99	2.747
310.71	19.87	8.88
323.58	4.11	0.371
324.79	7.96	0.671
332.77	1.19	0.214
336.78	0.54	1.06
338.48	1.46	0.881
345.46	22.21	0.621
362.22	2.57	0.682
368.51	2.37	0.56
378.89	2.14	0.785
391.47	1.56	1.048
399.8	11.91	1.327
402.43	47.45	0.853
417.35	7.69	0.504
422.04	16.19	0.142
423.75	49.09	0.936
433.63	1.27	2.197
437.3	12.82	0.702
455.08	8.93	1.024
460.06	17.88	0.29
467.37	3.11	0.272
475.03	52.99	0.276
486.35	3.41	0.201
493.83	30.71	0.858
497.8	14.23	0.176
505.37	40.83	0.383
508.45	4.5	0.18
510.63	4.46	0.279
514.3	13.24	0.027
519.5	6.18	0.195
523.05	22.47	0.236
529.18	38.69	0.202
533.91	40.42	0.088
545.56	50.41	0.304
548.32	39.27	0.842
560.92	46.23	0.987

563.06	14.26	0.172
571.41	22.05	0.479
585.6	11.08	0.187
593.67	7.81	6.218
609.62	22.88	0.494
611.57	5.61	0.303
619.11	23.91	0.157
627.56	0.45	0.088
635.77	10.11	0.354
641.25	3.15	0.38
656.71	17.54	0.113
660.54	4.77	0.05
668.12	45.97	0.347
673.52	5.98	0.264
675.89	3.16	0.336
684.5	7.68	0.389
689.47	5.88	2.148
696.39	57.45	1.045
701.33	73.57	2.96
704.41	33.62	2.067
705.45	155.32	0.161
710.7	185.98	0.153
710.94	98.01	0.371
726.76	31.47	0.523
733.99	37.04	0.183
738.76	5.93	1.228
746.27	26.71	0.582
772.58	66.2	0.903
773.88	43.52	0.277
780.27	89.57	0.275
781.63	85.55	0.649
784.86	114.84	0.428
789.02	78.82	0.402
794	26.01	2.056
797.04	76.51	0.732
823.3	28.39	0.169
829.1	1.27	0.062
840.23	0.11	0.722
882.28	4.29	0.064
888.49	6.7	0.085
898.14	1.54	0.219
922.07	1.88	0.012
924.16	2.43	0.024
968.67	19.31	0.033
975.7	17.34	0.037
981.72	2.69	0.138
996.48	11	0.052
1.00E+03	60.56	0.122
1.01E+03	35.66	0.222
1.03E+03	31.51	0.153
1.04E+03	24.08	0.198
1.07E+03	13.86	0.086
1.08E+03	100.56	0.006
1.10E+03	85.04	0.043
1.10E+03	39.76	0.119
1.11E+03	207.78	0.26
1.13E+03	24.64	0.174
1.13E+03	21.96	0.489
1.14E+03	222.19	0.112
1.15E+03	17.95	0.046

1.16E+03	24.61	0.066
1.17E+03	13.46	0.346
1.18E+03	852.03	0.194
1.22E+03	57.82	0.016
1.23E+03	386.9	0.037
1.23E+03	59.53	0.028
1.24E+03	118.48	0.027
1.25E+03	212.05	0.039
1.26E+03	70.61	0.07
1.28E+03	728.1	0.007
1.28E+03	343.25	0.408
1.32E+03	617.82	0.244
1.33E+03	523.84	0.106
1.34E+03	655.13	0.101
1.34E+03	447.42	0.065
1.37E+03	239.9	0.126
1.37E+03	14.9	0.159
1.38E+03	206.11	0.1
1.38E+03	9.52	0.146
1.44E+03	1.14E+03	0.021
1.45E+03	310.79	0.08
1.76E+03	47.88	2.048
1.78E+03	110.72	0.467
1.80E+03	426.8	0.554
1.83E+03	1.97E+03	0.229

Table S2: Vibrational Analysis of the systems $I_2/B_{24}N_{24}$.

**Frequency (1/cm)**	**Intensity IR (km/mol)**	**Intensity Raman (km/mol)**
40.81	0.15	102.148
52.66	6.56	777.117
59.31	4.95	321.028
63.94	5.55	705.092
79.69	1.02	54.123
97.45	0.07	19.123
106.6	0.45	5.46
123.78	1.14	5.911
139.95	3.06	6.326
145.17	0.64	5.969
153.65	2.25	17.765
157.54	0.36	25.881
169.01	0.92	9.41
187.82	0.29	16.441
205.98	0.96	8.854
220.53	1.79	28.951
231.33	6.83	54.491
241.35	0.87	1.257
251.38	9.79	74.171
254.14	1.32	12.812
272.76	1.66	9.6
284.61	4.06	3.776
291.65	8.14	1.472
295.68	2.22	6.591
300.66	9.55	7.018
306.11	3.06	4.246
310.54	5.5	4.138
319.57	5.46	11.647
329.4	1.62	1.289
333.93	0.44	3.852
339.09	4.74	1.389
347.87	3.17	1.023
356.75	13.76	4.128
364.47	5.32	1.416
370.8	2.82	1.435
376.81	0.11	8.805
386.17	6.19	10.516
399.68	0.43	0.512
410.22	3.59	3.208
418.35	9.66	10.856
426.88	8.26	2.759
431.4	63.48	23.551
437.78	18.96	3.556
445.69	21.25	1.609
455.9	45.01	7.708
460.95	24.04	3.19
466.97	22.53	3.132
481.58	3.8	0.578
483.78	15.34	2.468
495.39	21.49	13.84
497.48	21.01	9.468
505.23	9.06	0.786
509.91	24.95	1.893
512.37	6.18	1.791
516.76	5.5	0.538
518.69	11.91	1.214
521.46	22.79	2.892
529.85	33.64	8.788
531.27	8.51	12.982

545.06	118.63	41.645
548.62	35.38	5.192
555.75	12.46	1.586
557.43	25.02	2.139
567.76	30.81	5.589
576.49	49.77	26.682
584.41	2.44	5.439
597.03	0.49	3.773
605.86	17.81	1.036
609.79	21.77	2.495
626.79	5.88	0.119
638.76	4.86	2.435
646.9	35.27	2.134
651.74	2.9	0.479
657.88	4.89	1.428
665.59	18.26	6.052
673.4	5.06	2.487
676.15	57.31	0.554
680.3	24.85	1.841
688.52	2.9	2.638
693.76	43.49	7.725
695.77	107.41	3.64
697.95	69.48	0.561
700.09	10.38	0.761
704.46	131.2	7.601
711.72	122.69	1.555
722.76	79.4	4.708
726.09	61.01	2.17
730.31	47.84	0.234
740.32	1.61	0.126
750.96	22.93	1.955
772.76	4.37	0.845
781.08	91.74	11.637
783.4	7.14	0.375
788.67	217.02	17.083
790.93	2.4	3.78
795.62	127.35	10.465
802.17	6.35	1.67
817.65	17.31	1.721
833.33	2.91	0.844
847.46	0.79	2.4
889.09	6.76	0.664
898	12.15	3.186
904.13	9.82	9.087
911.49	4.73	3.032
937.4	6.83	0.485
941.26	2.39	0.645
979.79	17.34	0.44
990.07	6.58	4.348
993.47	14.08	1.103
1.01E+03	35.7	6.052
1.02E+03	20.45	0.687
1.04E+03	52.79	3.504
1.05E+03	24.05	10.378
1.07E+03	3.91	6.669
1.09E+03	34.5	11.565
1.10E+03	36.76	3.018
1.11E+03	48.47	9.507
1.12E+03	35.08	0.656
1.14E+03	110.75	13.704

1.14E+03	102.38	4.244
1.15E+03	114.41	8.184
1.16E+03	170.48	9.044
1.17E+03	55.99	0.734
1.19E+03	7.38	1.188
1.20E+03	244.09	57.727
1.21E+03	225.81	4.098
1.22E+03	115.57	0.559
1.24E+03	324.48	5.574
1.25E+03	78.15	6.596
1.26E+03	95.82	18.456
1.26E+03	324.01	5.976
1.28E+03	15.74	4.718
1.29E+03	466.82	52.478
1.30E+03	358.72	17.495
1.33E+03	286.51	5.121
1.35E+03	314.74	13.703
1.36E+03	71.53	20.418
1.37E+03	295.11	26.973
1.39E+03	217.18	5.549
1.41E+03	91.43	23.857
1.42E+03	359.8	21.148
1.44E+03	703.18	27.066
1.48E+03	97.84	1.63
1.78E+03	165.17	19.242
1.81E+03	24.31	3.789
1.82E+03	30.77	9.256
1.84E+03	781.78	68.734

Table S3: Vibrational Analysis of the systems $I_2/B_{24}N_{24}@Mg^{2+}$.

**From**	**To**	**TD-ex[eV]**	**KS-ex[eV]**	**TD-ex[nm]**	**KS-ex[nm]**	**[Ha]**	**f_osc**
HOMO	LUMO-	4.25	4.27	292	291	0.156207	0.000000
HOMO-1	LUMO+	4.25	4.27	292	291	0.156257	0.000000
HOMO	LUMO+	4.29	4.27	289	290	0.157714	0.003411
HOMO-1	LUMO-	4.29	4.27	289	290	0.157765	0.003463
HOMO	LUMO+1-	4.50	4.50	276	275	0.165278	0.003308
HOMO-1	LUMO+1-	4.50	4.51	276	275	0.165323	0.003289
HOMO	148-	4.59	4.51	270	275	0.168822	0.005800
140	LUMO-	4.72	4.67	263	266	0.173379	0.007334
139	LUMO+	4.72	4.67	263	266	0.173428	0.007323
141	LUMO-	4.75	4.78	261	259	0.174663	0.050667
139	LUMO+2+	4.95	4.91	251	253	0.181776	0.012384
135	LUMO+	5.04	5.02	246	247	0.185345	0.002741
136	LUMO+	5.04	5.02	246	247	0.185350	0.003125
138	LUMO+2+	5.06	5.02	245	247	0.185824	0.010157
138	148+	5.06	5.02	245	247	0.185889	0.009080
138	LUMO+1-	5.14	5.14	241	241	0.188818	0.139794
HOMO	151-	5.19	5.16	239	240	0.190633	0.004675
HOMO-1	151-	5.19	5.17	239	240	0.190677	0.004214
132	LUMO-	5.28	5.26	235	236	0.193995	0.002773

Table S4: Optical Absorption Analysis of the system $B_{24}N_{24}$. The HOMO corresponds to Kohn-Sham state 144 while the LUMO corresponds to state 145.

**From**	**To**	**TD-ex[eV]**	**KS-ex[eV]**	**TD-ex[nm]**	**KS-ex[nm]**	**[Ha]**	**f_osc**
HOMO	LUMO+	2.11	2.18	588	568	0.077457	0.000000
HOMO	LUMO-	2.35	2.18	527	568	0.086397	0.000131
HOMO-1	LUMO-	2.52	2.59	492	478	0.092635	0.000000
HOMO-1	LUMO+	2.74	2.59	452	478	0.100876	0.005351
HOMO	LUMO+1+	2.89	2.91	429	426	0.106086	0.000000
HOMO	LUMO+1-	2.96	2.91	419	426	0.108734	0.007053
194	LUMO-	3.12	3.08	397	403	0.114752	0.001736
HOMO-2	LUMO-	3.14	3.08	395	403	0.115421	0.007628
HOMO-1	LUMO+1-	3.36	3.32	369	373	0.123489	0.001091
193	LUMO+	3.44	3.42	360	363	0.126508	0.003252
HOMO	201-	3.55	3.54	349	350	0.130637	0.001283
HOMO	202-	3.57	3.54	347	350	0.131151	0.002797
191	LUMO-	3.58	3.57	347	348	0.131495	0.014285
HOMO-1	LUMO+2+	3.73	3.71	332	334	0.137091	0.002083
187	LUMO+	3.78	3.76	328	330	0.138747	0.020050
185	LUMO+	3.79	3.80	327	326	0.139139	0.007428
HOMO	203-	3.83	3.80	324	326	0.140745	0.020258
194	LUMO+1+	3.85	3.80	322	326	0.141385	0.003758
HOMO-2	LUMO+1+	3.86	3.80	322	326	0.141703	0.010796
HOMO-1	201-	3.96	3.95	313	314	0.145686	0.007286
HOMO	204-	3.97	3.95	312	314	0.146048	0.001403
184	LUMO-	3.98	3.96	312	313	0.146151	0.001075
HOMO-1	202+	3.99	3.98	311	311	0.146571	0.004207
HOMO	205-	4.03	4.01	308	309	0.147940	0.002025
182	LUMO-	4.10	4.07	303	304	0.150533	0.023020
181	LUMO-	4.13	4.12	300	301	0.151633	0.002792
HOMO-1	203+	4.20	4.17	295	297	0.154487	0.001216
191	LUMO+1-	4.21	4.18	295	297	0.154604	0.021723
HOMO-2	LUMO+2-	4.23	4.20	293	295	0.155420	0.001850
194	LUMO+2-	4.23	4.20	293	295	0.155466	0.001439
190	LUMO+1-	4.26	4.23	291	293	0.156383	0.002032
180	LUMO+	4.28	4.30	290	289	0.157240	0.005432
189	LUMO+1-	4.33	4.30	287	289	0.158996	0.000462
HOMO	208-	4.41	4.40	281	282	0.161950	0.003536
188	LUMO+1+	4.45	4.44	279	279	0.163401	0.014101
HOMO-2	201+	4.45	4.44	278	279	0.163673	0.003904
194	202+	4.46	4.44	278	279	0.163915	0.001292
186	LUMO+1+	4.47	4.44	277	279	0.164367	0.001231
HOMO-2	202-	4.50	4.49	276	276	0.165232	0.012259
194	201+	4.50	4.49	276	276	0.165310	0.009893
HOMO	210+	4.51	4.51	275	275	0.165602	0.000000
185	LUMO+1+	4.51	4.51	275	275	0.165886	0.002675
HOMO	209+	4.53	4.52	274	275	0.166304	0.005343
HOMO	209+	4.53	4.52	274	275	0.166357	0.004306
HOMO	210+	4.57	4.58	271	271	0.168084	0.003717
HOMO	211-	4.60	4.59	269	270	0.169088	0.002742
190	LUMO+2-	4.62	4.60	268	270	0.169719	0.001051
HOMO-1	206-	4.64	4.65	267	267	0.170446	0.006771
HOMO-2	203-	4.68	4.67	265	266	0.171937	0.001548
184	LUMO+1+	4.69	4.67	265	266	0.172201	0.002729
193	202-	4.69	4.67	264	266	0.172437	0.002457
189	LUMO+2-	4.70	4.67	264	266	0.172633	0.006328
183	LUMO+1-	4.71	4.71	263	263	0.172988	0.006702
HOMO	213-	4.77	4.76	260	260	0.175437	0.001207
HOMO-1	207+	4.79	4.78	259	259	0.175933	0.003281
187	LUMO+2-	4.80	4.78	258	259	0.176283	0.002755
HOMO-1	208-	4.81	4.78	258	259	0.176651	0.007351
HOMO-1	208+	4.81	4.78	258	259	0.176704	0.008183
191	202-	4.83	4.81	257	258	0.177347	0.004978

178	LUMO+	4.84	4.85	256	256	0.177977	0.001109
190	201-	4.86	4.86	255	255	0.178590	0.001413
HOMO-2	204-	4.87	4.86	255	255	0.178788	0.001296
190	202-	4.87	4.86	255	255	0.178947	0.001519
194	204+	4.87	4.87	255	254	0.178999	0.001102
185	LUMO+2-	4.89	4.87	254	254	0.179599	0.001008
194	205-	4.91	4.91	252	252	0.180571	0.001810
190	202+	4.91	4.92	252	252	0.180612	0.015348
HOMO-2	205-	4.93	4.92	252	252	0.181040	0.005843
189	201-	4.93	4.92	252	252	0.181146	0.001410
HOMO	214+	4.94	4.93	251	252	0.181674	0.006570
HOMO	214-	4.95	4.93	251	251	0.181748	0.008750
HOMO-1	209+	4.96	4.93	250	251	0.182431	0.006916
180	LUMO+1-	4.97	4.97	249	250	0.182657	0.002505
189	202-	4.98	4.98	249	249	0.182933	0.015722
HOMO-1	210-	4.99	4.99	249	248	0.183202	0.002432
192	203-	5.01	5.01	248	247	0.184039	0.013617
191	203+	5.02	5.01	247	247	0.184385	0.001724
196	211+	5.02	5.02	247	247	0.184607	0.001863
196	211-	5.03	5.02	247	247	0.184676	0.002251
184	200-	5.05	5.04	246	246	0.185454	0.003399
177	LUMO+	5.06	5.06	245	245	0.185871	0.015113
187	202+	5.06	5.06	245	245	0.186073	0.002004
187	202-	5.07	5.07	244	244	0.186417	0.001093
183	200+	5.08	5.07	244	244	0.186794	0.001695
195	206-	5.10	5.08	243	244	0.187271	0.004179
194	206+	5.10	5.08	243	244	0.187334	0.002049
HOMO	216+	5.11	5.10	243	243	0.187710	0.006542
190	203+	5.11	5.11	243	243	0.187789	0.001338
186	201-	5.12	5.11	242	243	0.187984	0.001086
186	202-	5.12	5.12	242	242	0.188108	0.008424
182	200+	5.14	5.14	241	241	0.188876	0.005290
185	201-	5.14	5.14	241	241	0.188982	0.029862
196	212-	5.16	5.15	240	241	0.189638	0.002540
189	203+	5.17	5.17	240	240	0.189972	0.003113
174	LUMO+	5.17	5.17	240	240	0.190058	0.005158
HOMO	217+	5.18	5.17	239	240	0.190249	0.001012
192	205-	5.20	5.19	238	239	0.191272	0.001719
196	213+	5.21	5.22	238	238	0.191457	0.002868
191	204-	5.22	5.24	238	237	0.191697	0.004051
181	200+	5.22	5.24	237	236	0.191992	0.018638
190	204-	5.29	5.29	234	234	0.194542	0.001190
184	201+	5.30	5.29	234	234	0.194706	0.002393

Table S5: Optical Absorption Analysis of the system $I_2/B_{24}N_{24}$. The HOMO corresponds to Kohn-Sham state 197 while the LUMO corresponds to state 198.
