# Supplementary figures and images for: Structural, Electronic and Vibrational Properties of B24N24 Nanocapsules: Novel Anodes for Magnesium Batteries
Source: Nanomaterials (Basel). 2024 Jan 26;14(3):271. doi: 10.3390/nano14030271 (PMC10856419; doi:10.3390/nano14030271)

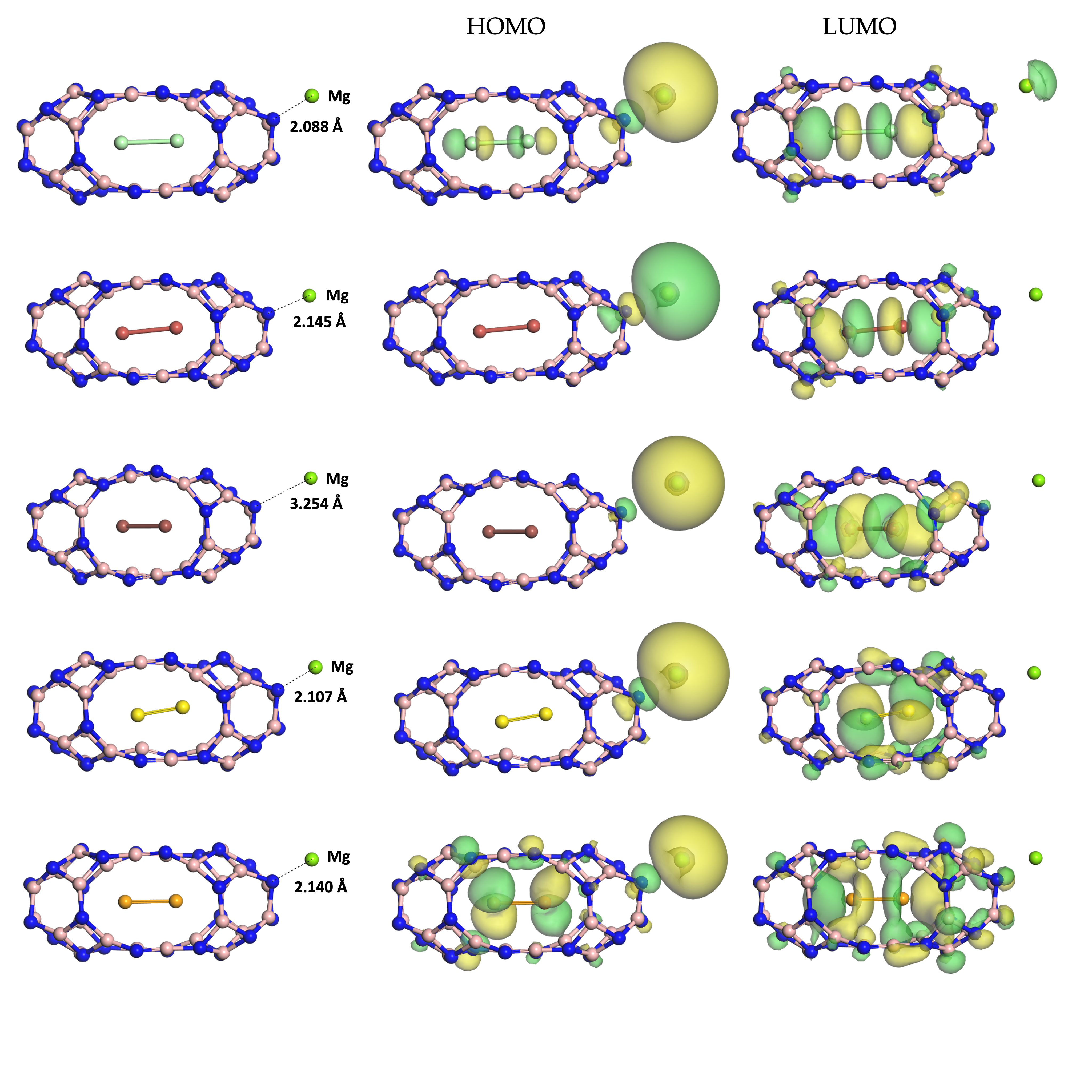

Supplement: Supplementary file 1 [file nanomaterials-14-00271-s001.zip › B24N24endonanoMg.png]

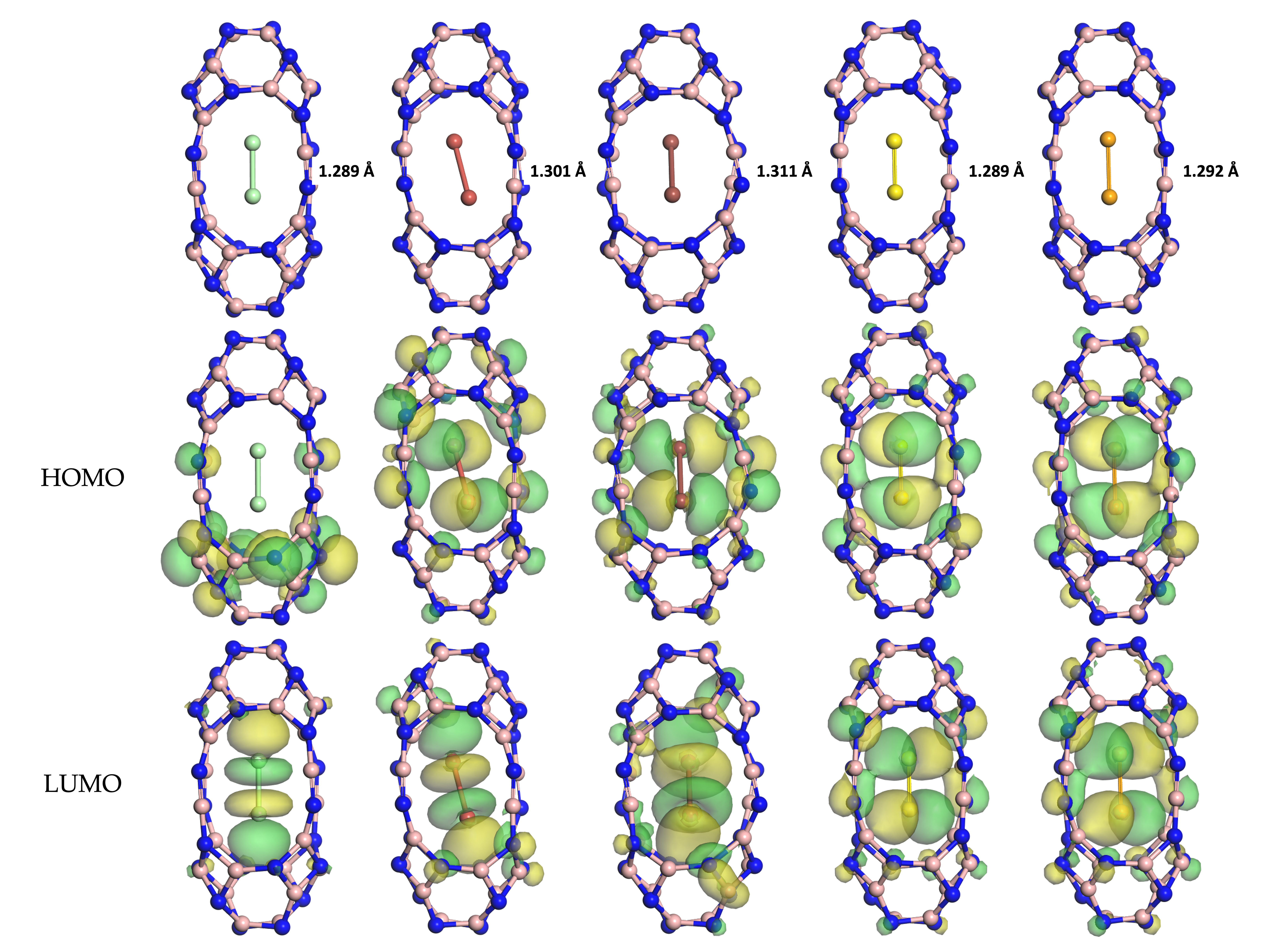

Supplement: Supplementary file 1 [file nanomaterials-14-00271-s001.zip › Endonanocapsules.png]

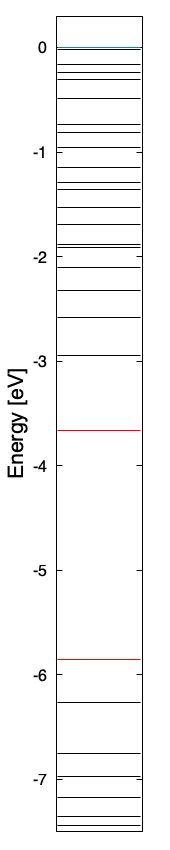

Supplement: Supplementary file 1 [file nanomaterials-14-00271-s001.zip › Energy_Levels_I2_B24N24.png]
